# Tumor apelin, not serum apelin, is associated with the clinical features and prognosis of gastric cancer

**DOI:** 10.1186/s12885-016-2815-y

**Published:** 2016-10-12

**Authors:** Meiyan Feng, Guodong Yao, Hongwei Yu, Yu Qing, Kuan Wang

**Affiliations:** 1 Department of Pathology, The Affiliated Tumor Hospital of Harbin Medical University, Harbin, 150081 China; 2 Department of Histology and Embryology, Harbin Medical University, Harbin, 150086 China; 3 Department of Gastrointestinal Surgery, The Affiliated Tumor Hospital of Harbin Medical University, 150 HaPing Road, Nangang District, Harbin, Heilongjiang Province 150081 China

**Keywords:** Apelin, Prognosis, Gastric cancer

## Abstract

**Background:**

To study the association between Apelin expression and the clinical features and postoperative prognosis in patients with gastric cancer (Int J Cancer 136:2388-2401, 2015).

**Methods:**

Tumor samples and matched adjacent normal tissues were collected from 270 patients with GC receiving surgical resection. The tumor and serum Apelin levels were determined by immunohistochemistry and ELISA methods, respectively. GC cell lines were cultured for migration and invasive assays.

**Results:**

Our data showed that tumor Apelin expression status, instead of serum Apelin level, was closely associated with more advance clinical features including tumor differentiation, lymph node and distant metastases. Moreover, patients with high tumor Apelin level had a significantly shorter overall survival period compared to those with low Apelin expression and those with or negative Apelin staining. Our in vitro study revealed that the Apelin regulated the migration and invasion abilities of GC cell lines, accompanied by up-regulations of a variety of cytokines associated with tumor invasiveness.

**Conclusion:**

Our data suggest that tumor Apelin can be used as a marker to evaluate clinical characteristics and predict prognosis in GC patients.

## Background

Gastric cancer is among the leading causes of global cancer-related mortality [1]. Despite of the recent advances in diagnosis and therapy, the prognosis of GC patients is still poor. Usually, the 5-year survival rates are less than 20 % [2–4]. Currently, there is no specific marker for early diagnosis and prognosis prediction, although a number of proteins have been previously reported to be associated with the outcome of GC patients [5–8].

Apelin is a member of the endogenous ligand of the human G protein receptor, known as APJ [9]. Both Apelin and APJ are extensively expressed in blood vasculature and stimulate angiogenesis by prompting endothelial cell growth [10–12]. Also, Apelin induces the maturation of tumor blood capillaries and prompts tumor vascularization [13]. Moreover, Apelin is upregulated in human cancers and its association with cancer outcomes were reported as well [14–17]. In addition, recent studies show that Apelin has lymphangiogenic potential and it is related to tumor growth and lymph node metastasis in vivo [18, 19].

However, the association of Apelin and gastric cancer remain largely unknown. A recent study reported a higher serum Apelin in patients with gastroesophageal cancer (GEC) compared to healthy controls [20]. Moreover, there is a weak positive correlation between serum Apelin concentrations and tumor Apelin expression levels [20]. In this study, we enrolled GC patients to further investigate the role of tumor and serum Apelin in the clinical features, in particular, disease characteristics and prognosis in GC patients.

## Methods

### Samples

Tumor samples and matched adjacent non-tumorous tissues were collected from 270 patients with GC receiving surgical resection between 1 January 2009 and 31 December 20013. None of the patients with carcinomas underwent either chemotherapy or radiotherapy before surgery. The tumor stage of patients was determined by the UICC-TNM classification. All the tissue samples were identified by clinical pathologist and then were fixed by formaldehyde and embedded by paraffin for further study. We also collected tissue samples from 81 patients with chronic gastritis as control. All patients were followed by consulting their documents, or through clinic visit or telephone interviews. Overall survival (OS) period was defined as the time interval between the date of surgery and date of death or last follow-up.

### Immunohistochemistry

GC tissues sections fixed by formalin and embedded by paraffin were dewaxed in xylene and rehydrated with gradient ethanol. The sections were incubated with rabbit anti-Apelin monoclonal antibody (1:150, Abcam, USA) at 4 °C overnight. The immune complex was detected by a standard avidin-biotin detection system (Dako, USA). The sections were evaluated by three pathologists who were blinded to clinicopathologic information. Apelin staining score = positive cell score + staining intensity score. The percentage of positive cells was classified by four grades (percentage scores): 0 [21], <1/3 [21], 1/3-2/3 [22] and >2/3 [22]. The intensity of staining was also divided into four grades (intensity scores): no staining [21], weak staining [21], moderate staining [22] and strong staining [22]. The overall scores 0, 1–2, 3–4, and 5–6 were defined as negative (−), weak positive (±), moderate positive (+), and strong positive (++) respectively.

### Serum apelin level detection

The peripheral blood samples were collected from all participants after 12-h overnight fast. The serum Apelin concentration was measured by an ELISA kit (Apelin-12, Phoenix pharmaceuticals, Belmont, USA) according to manufacturer’ protocol. The sensitivity was 0.05 ng/mL, and intra- and inter-assay variations were <5 and <10 %, respectively.

### Cell lines and cell culture

Three GC cell lines, namely, SGC-7901, MKN-45, AGS and an immortalized normal gastric epithelial cell line GES-1, were purchased from Cell Bank of Type Culture Collection (Shanghai China). Cells were maintained in Dulbecco’s Modified Eagle’s medium (DMEM, Gibco) containing 10 % fetal bovine serum (FBS), 100 U/ml penicillin, and 100 ug/ml streptomycin.

### Gene silencing of APJ with siRNA

GC cell lines were transfected with 200 nmol/L APJ or nonspecific siRNA (Ambion, USA) in culture medium for 48 h. The medium was then replaced with fresh DMEM and the cells were incubated at 37 °C for an additional 24 h. The cells were collected and stored at −80 °C until assayed for protein expression by Western blotting as detailed below.

### Proliferation assay

The effect of hypoxia on the viability of cultured cells was evaluated by 2-(2-methoxy-4-nitrophenyl)-3-(4-nitrophenyl)-5-(2,4-disulfophenyl)-2H-tetrazolium, a monosodium salt (WST-8) assay (Dojindo Molecular Technologies, Japan). Briefly, cells are treated with Apelin (50 and 100 ng/mL) and seeded (cell density of 5 × 10^3^ per well) in 96-well microplates and cultured in the hypoxic incubator for 8 h, followed by addition of 10 ul WST-8 solution to each well. After 4 h of incubated at 37 °C, absorbance was measured at 450 nm using a microplate reader (Benchmark Microplate Reader, BIO-RAD) with a reference wavelength of 490 nm.

### Cell migration and invasion analysis

Cells were treated with Apelin (50 and 100 ng/mL) for cell migration and invasion assay by using Transwell chamber (Corning, NY, USA), which coated with Matrigel (BD Bioscience) in invasion assays. 5 × 10^4^ cells were collected and seeded in the upper chamber without serum. 10 % fetal bovine serum was used as a chemoattractant in lower chamber. After 8 h of incubation, cells that did not invade through the pores were wiped out with cotton wool. Invaded cell was stained with 20 % methanol and 0.2 % crystal violet and counted with an inverted microscope (Olympus, Japan).

### Western blot analysis

Cells were lysed with RIPA lysis buffer and the lysates protein concentration was measured by a BCA Protein Assay Kit (Pierce, Rockford, USA). The protein samples (10 μg/well) were loaded onto 10 % SDS-PAGE and then transferred onto PVDF membranes. After blocked by skim milk, the membranes were incubated in the primary antibodies for overnight at 4 °C and then in the HRP-conjugated secondary antibody for 2–3 h at room temperature. The primary antibodies used in the experiments were anti-Apelin, anti-APJ (both 1:1000; Abcam, USA), anti- Matrix metalloproteinases1 (MMP1) and MMP9 (both 1:1000; Santa Cruz, USA), anti-Bone morphogenetic protein 2 (BMP2, 1:1000; Santa Cruz, USA), anti-interleukin1 and 6 (IL1 and IL6, both1:1000; Santa Cruz, USA), Finally the protein band images were captured by ECL reagent (Thermo, USA).

### Statistical analysis

All data were analyzed using the SPSS 19.0 software (SPSS Inc., Chicago, USA) and GraphPad Prism (Version 6.02 for Windows, Graphpad Software, USA). Qualitative variables were analyzed using either the Chi Square Test or the Fisher’s test. Correlations between Apelin expression and clinical features of GC patients were determined by chi-square test. Survival analysis was performed using the Kaplan-Meier method. COX analysis was used to determine the independent prognostic factor for GC patients. Unless otherwise noted, *P* < 0.05 was accepted as significant.

## Results

The demographical information of patients with GC and patients with chronic gastritis listed in Table 1. There is no significant difference in mean age, gender distribution, smoking status and Helicobacter Pylori infection status between two groups.

**Table 1:** The demographical data of patients with GC cancer and chronic gastritis

Variables	Patients with GC	Patients with chronic gastritis	*P* value
Age (years)	55.6 ± 5.4	55.5 ± 6.3	0.236
Gender			
Male	144	35	0.109
Female	126	46	
Smoking status			
Non-smoker	57	15	0.156
Ever-smoker	101	23	
Current-smoker	112	33	
Hp infection			
Presence	187	57	0.87
Absence	83	24	

*HP* Helicobacter Pylori

**Fig. 1:**
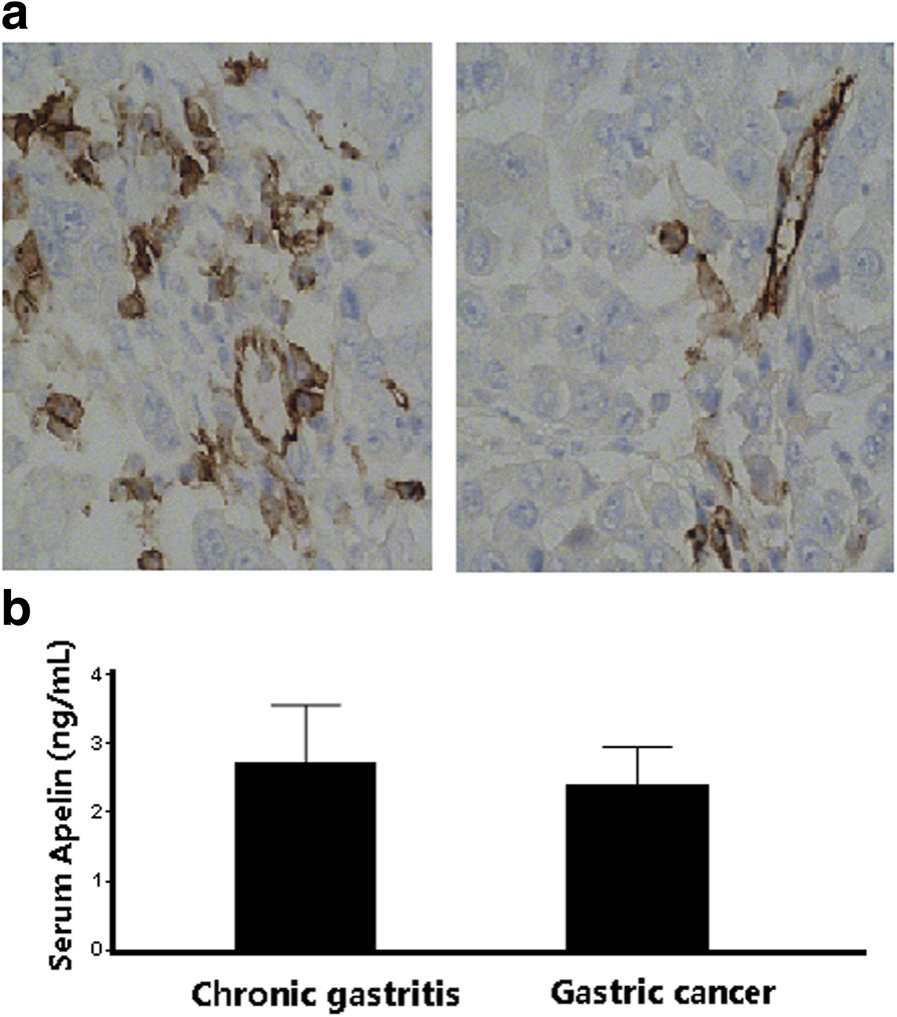
**a** shows the representative images about Apelin immunohistochemical stainings. Left, high Apelin expression sample. Apelin is expressed in cytoplasma and also in vascular endothelial cells in the tumor tissue. Right, low Apelin expression sample, Apelin are dominately expressed in vascular endothelial cells. **b** The serum Apelin levels between patients with GC and chronic gastritis. There is no significant difference in serum Apelin levels between two groups (2.84 ± 1.13 vs.2.52 ± 0.78, ng/mL, *P* = 0.453)

The representative images about Apelin immunohistochemical stainings are shown in Fig. 1a. Apelin is expressed in cytoplasma and also in vascular endothelial cells in the tumor tissue. Cytoplasmic Apelin staining was identified in 112 of 270 normal gastric mucosa samples and 36 of 81 samples with chronic gastritis (41.2 % vs.44.4 %, *P* = 0.635). The GC patients with strong Apelin staining (show as “++” in Table 1) are 120, with moderate Apelin staining are 99 (show as “+” in Table 1), and only 51 patients had weak or no Apelin staining in this group (show as “±/-” in Table 1). There are a significant difference in Apelin expression status between patients with GC and with chronic gastritis (Table 2).

**Table 2:** The APELIN expression status among GC cancer samples, noncancerous tissues and samples from Chronic gastritis

APELIN Expression level	Gastric cancer	Adjacent normal tissue	Chronic gastritis	*P* value
++	120	48	25	<0.001
+	99	89	11	
±/-	51	133	45	

In contrast, the serum Apelin levels remains similar among GC and Chronic gastritis groups (2.84 ± 1.13 vs.2.52 ± 0.78, ng/mL, *P* = 0.453, Fig. 1).

We next investigated the relationship between tumor Apelin expression status and clinical characteristics of GC patients. As shown in Table 3, high expression of Apelin in GC cancer samples was associated with poor differentiation, tumor stage, lymph node metastases, and distant metastases. However, there were no significant associations between Apelin expression levels and gender, age, or histology type and tumor size was found (Table 3). When the serum Apelin is studied, we only found that GC patients with lymph node metastasis had a higher serum Aplein level compared to those without (*P* = 0.043). However, serum Apelin levels are not associated with the other clinical characteristics in GC patients (All *P* > 0.05, Table 3).

**Table 3:** The association between Tumor and serum Apelin levels and clinical characteristics in GC patients

Variables	Number of cases	Apelin expression level			*P* value	Serum Apelin (ng/mL)	*P* value
		++ (*n* = 120)	+(*n* = 99)		-(*n* = 51)		
Gender							
male	175	76	69	30	0.377	3.35 ± 1.45	0.345
female	95	44	30	21		3.33 ± 1.37	
Age (years)							
< 60	171	80	60	31	0.569	3.31 ± 1.76	0.143
> 60	99	40	39	20		3.35 ± 1.77	
Tumor size (cm)							
< 5	91	39	29	23	0.142	3.29 ± 1.67	0.167
> 5	179	81	70	28		3.38 ± 1.26	
Tumor differentiation							
Poorly	166	98	58	10	<0.001	3.24 ± 2.01	0.187
Well, moderately	104	22	41	41		3.36 ± 1.555	
Tumor stage							
T1 + T2	106	13	59	34	<0.001	3.40 ± 2.11	0.057
T3 + T4	164	107	40	17		3.27 ± 1.25	
Lymph node metastasis							
Negative	90	26	24	40	<0.001	3.27 ± 1.62	0.043
Positive	180	94	75	11		3.39 ± 1.28	
Distant metastasis							
Positive	88	51	21	16	0.004	3.34 ± 1.87	0.787
Negative	182	69	78	35		3.34 ± 0.87	

**Fig. 2:**
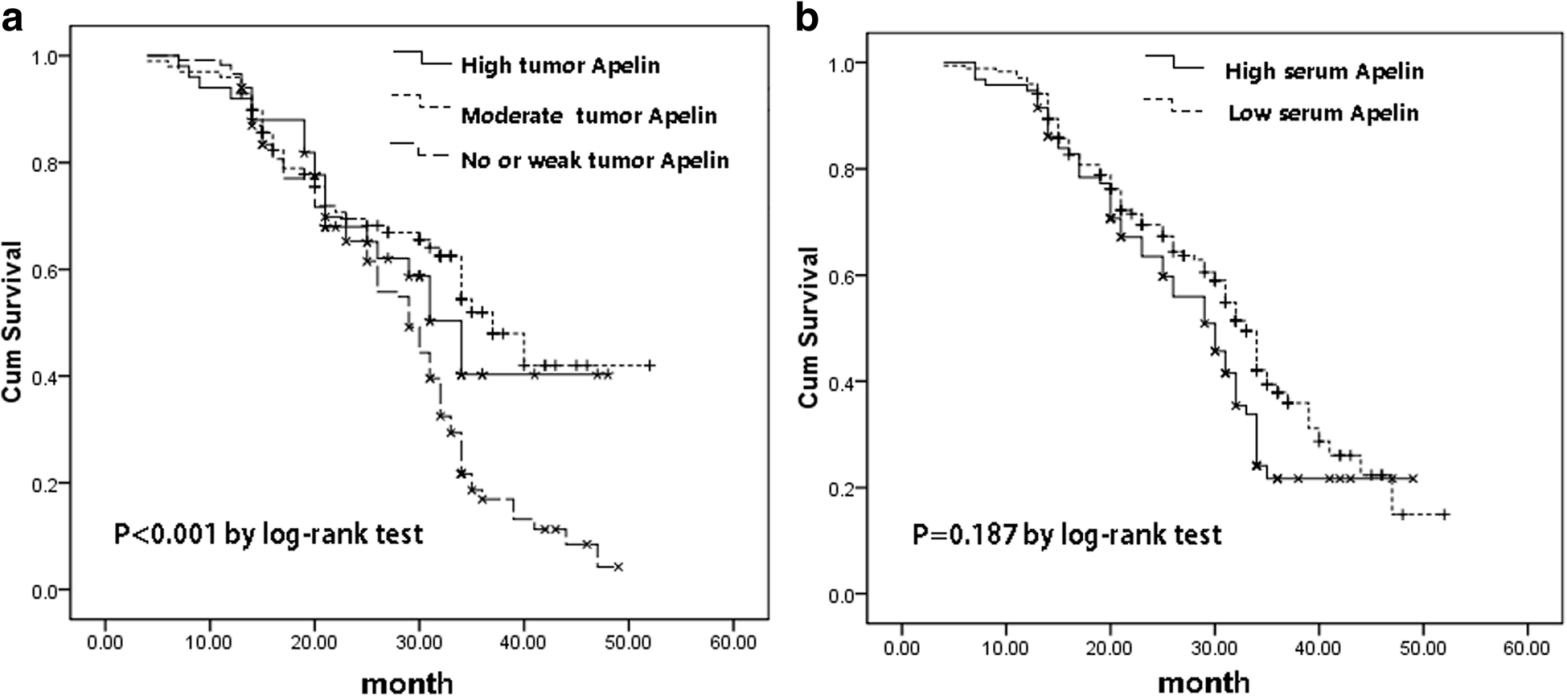
The relation of tumor Apelin expression status with the survival of GC patients by Kaplan-Miere curves. **a** Patients with strong Apelin staining had significantly shorter overall survival period 22.6 ± 4.9 months) compared to those with low Apelin expression (29.1 ± 3.7, months) and those with weak or negative Apelin staining (30.4 ± 6.4 months). **b** GC patients with high and low serum Apelin had similar overall survival period (26.9 ± 5.2 vs. 26.4 ± 2.9, months, *P* = 0.187 by log-rank test, Fig. 2b)

We further analyzed the relation of tumor Apelin expression status with the survival of GC patients in this study. As shown in Fig. 2a, patients with high tumor Apelin staining had a significantly shorter overall survival period compared to those with low Apelin expression and those with weak or negative Apelin staining (22.6 ± 4.9, 29.1 ± 3.7 and 30.4 ± 6.4, months, *P* < 0.001 by log-rank test, Fig. 2a). We used the mean serum Apelin value (2.84 ng/mL) as a cut-off value to subgroup all GC patients: those with equal or higher than 2.84 ng/mL were assigned into high serum Apelin group (*n* = 160) and those with lower than 2.84 ng/mL were assigned into low serum Apelin group (*n* = 110). We found these two groups had similar overall survival period (26.9 ± 5.2 vs. 26.4 ± 2.9, months, *P* = 0.187 by log-rank test, Fig. 2b).

Subsequently, as shown in Table 4, the univariate COX analysis revealed that the prognosis of GC patients were associated with lymph node metastasis (*P* = 0.004), tumor differentiation (*P* = 0.034) and tumor Apelin expression (*P* = 0.002), but not with serum Apelin level (*P* = 0.332). Furthermore, the multivariate mode of COX analysis revealed that tumor Apelin expression level wasn a independent prognostic factor for the overall survival in GC patients (*P* = 0.003).

**Table 4:** The Cox analysis of prognostic factors for GC patients

Univariate COX analysis			Multivariate COX analysis			
Variable	HR	95 % CI	*P*-value	HR	95 % CI	*P*-value
Lymph node metastasis	2.43	1.44–4.67	0.004	1.67	1.23–3.87	0.024
Tumor differentiation	1.59	1.17–2.89	0.034	1.53	1.10–2.98	0.021
Tumor APJ level	2.23	1.451–4.14	0.002	2.15	1.33–4.32	0.003

**Fig. 3:**
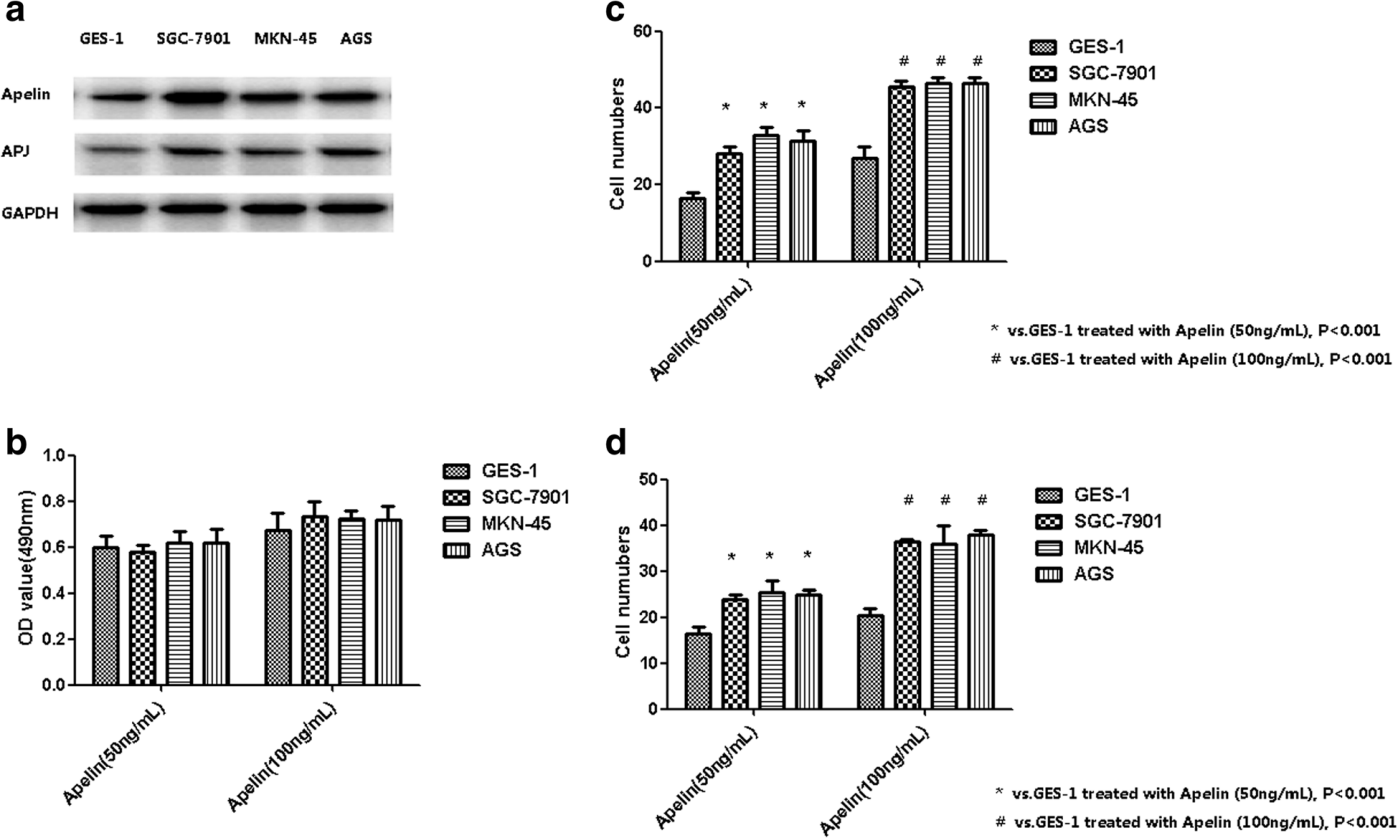
**a** SGC-7901, MKN-45 and AGS had a higher Apelin expression levels compared GES-1 by western blot assay. **b** Apelin treatment (50 and 100 ng/mL) for 8 h did not affect the proliferation rates in GC cell lines and non-cancer cell line GES-1. **c** and **d** The migration and invasion abilities of GC cell lines were significantly increased by Apelin treatment (50 and 100 ng/mL for 8 h, respectively)

In our in vitro study, we observed that all GC cell lines, including SGC-7901, MKN-45 and AGS had a 1.5 to 2 folds higher expression levels of Apelin compared to non-cancer cell line GES-1 (Fig. 3a). Similarly, the APJ expression level is higher in GC cell lines than in normal cell line GES-1 (Fig. 3a).

When these cells are treated with Apelin (50 and 100 ng/mL) for 8 h, we observed that proliferation rates remain similar between GC cell lines and non-cancer cell line GES-1 (Fig. 3b). However, the migration and invasion abilities of GC cell lines were significantly increased by Apelin treatment (Fig. 3c-d).

**Fig. 4:**
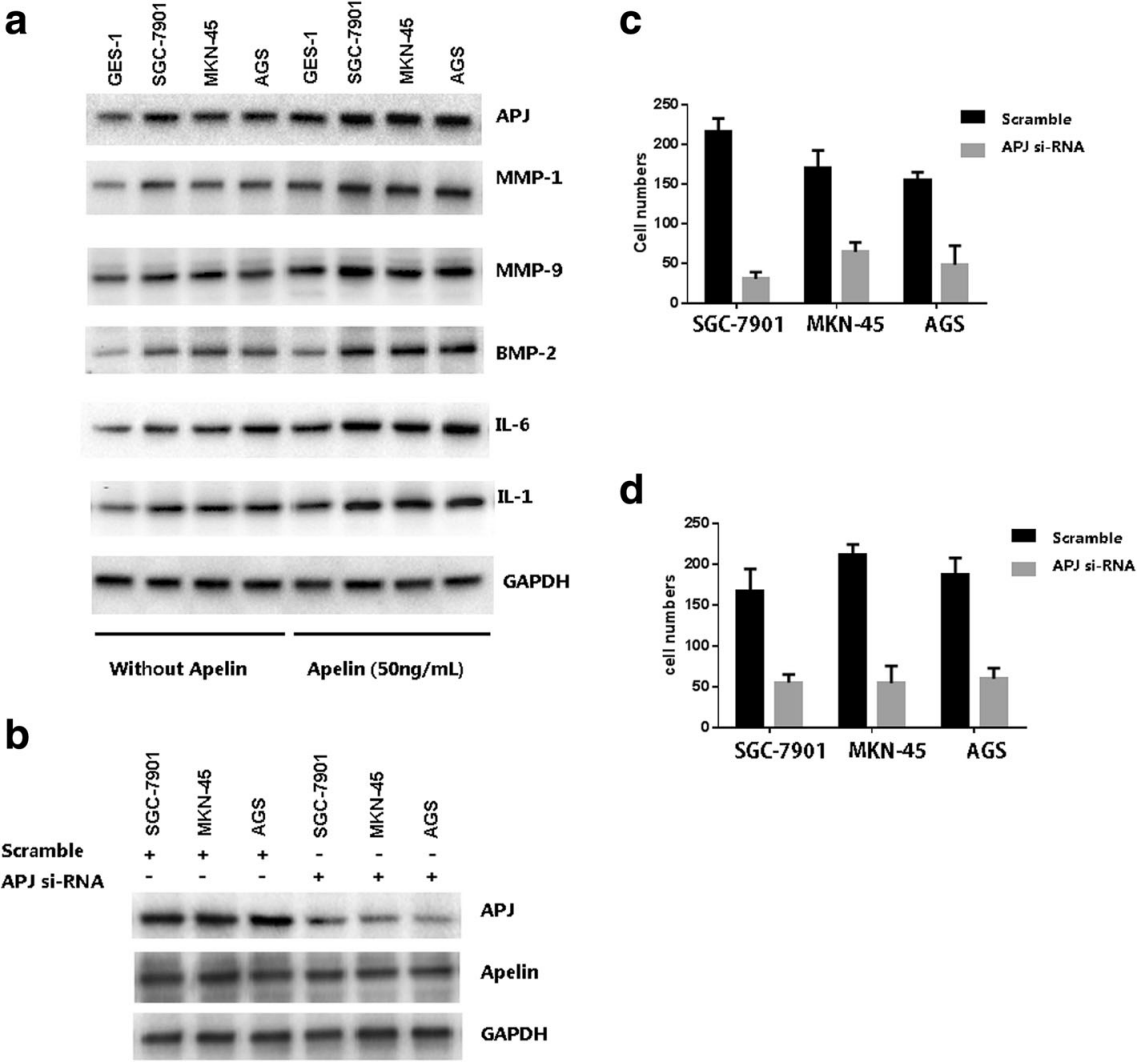
**a** Apelin treatment increases several cytokines known to facilitate tumor metastasis and progression. **b** APJ si-RNA reduces APJ in GC cell lines, without affecting Apelin expression. **c** and **d**. APJ siRNA transfection dramatically reduced the migration and invasion abilities in GC cell lines

Notably, we observed that Apelin treatment induced the protein expression of a variety of cytokines, such as APJ, MMP1, MMP9, BMP-2, IL1 and IL6 (Fig. 4a). All these cytokines are reported associated with tumor invasive or metastasis.

When these cells are transfected with Apelin receptor APJ si-RNA, an 85 % reduction of APJ was observed and Apelin expression was not affected (Fig. 4b). When the scramble and si-APJ RNA transfected cells were treated with Apelin (100 ug/mL for 24 h), We observed that there is a reduced migration and invasion abilities in GC cell lines (Fig. 4c and d, respectively).

## Discussion

In the present study, we studied the correlation between tissue and serum Apelin level with the clinical characteristics and prognosis of GC patients. Our data show that tissue Apelin expression status, instead of serum Apelin level, is closely associated with more advance clinical features and poorer outcome. Our in vitro data further reveals that GC cell lines over-expression Aplein and its receptor APJ, together with the other cytokines which are known to facilitate tumor metastasis and progression, including IL-1, IL6, MMP1, MMP9 and BMP-2. The inhibition of Apelin receptor APJ, reduces the cellular migration and invasion abilities in vitro. Our data suggest that tumor Apelin is a protein marker to evaluate the clinical features and to predict post-operative prognosis in GC patients.

Apelin is a peptide expressed in various tissues, including gastrointestinal tract, heart, lung, liver, and bone [23]. Previous experimental and clinical studies suggest that Apelin is a mitogenic factor for the endothelial cells and stimulates tumor angiogenesis. Recent studies show that Apelin was found to be up-regulated in a variety of human cancers. The Apelin/APJ pathway induces arteriogenesis in samples of poorly-differentiated hepatocellular carcinoma (HCC) [24]. Using Apelin as a marker to monitor tumor vessel normalization window during anti-angiogenic therapy was reported [17]. Co-expression of Apelin and APJ in tumor is the basis of an autocrine loop involved in the growth of colon adenocarcinomas [25]. A clinical study showed that Apelin up-regulation is associated with a poor prognosis in oral squamous cell carcinoma patients [24]. However, the role of Apelin in GC is not adequately studied to date.

A recent study detected the serum Apelin level in gastroesophageal cancer (GEC) patients and found that serum Apelin was significantly higher in cachectic patients than in the controls. Serum Apelin is positively correlated with hypersensitive C reactive protein level, suggesting that suggest that Apelin production in serum is probably related to systemic inflammatory response in GEC patients [20]. However, this study did not investigate the prognostic role of Apelin in GC patients. Given serum marker could be easily affected by external condition, such as inflammation and stress, it is of interest to study the effect of tumor Aplein in tumor tissues in GC patients. In our study, we found that GC patients had a significantly higher percentage of having strong Apelin staining than samples from chronic gastritis. However, the serum Apelin levels remains similar among GC and Chronic gastritis groups. Moreover, high expression of Apelin in GC cancer samples was associated with poor differentiation, lymph node metastases and distant metastases. However, serum Apelin levels are not associated with the other clinical characteristics in GC patients.

In this study, we detected several cytokines, including IL-1, IL6, MMP1, MMP9 and BMP-2. There factors are known to be correlated with tumor invasiveness and metastasis in gastric cancer [25–29]. We observed GC cell lines had a higher expression of these factor and their expression can be further increased by Apelin treatment. We postulate that Apelin may prompt tumor invasiveness through up-regulation of these factors.

Recent animal studies indicated that lymphatic vessels interact extensively with malignant cells. Moreover, lymphangiogenesis is associated with lymph node metastasis. Apelin overexpression induces intratumoral lymphangiogenesis and promotes lymphatic metastasis. Apelin increases lymphatic endothelial cells (LEC) spheroid numbers and stimulates capillary-like cord formation of LECs in vitro and promotes the growth of lymph vessels [18]. Consistent with these findings, in this study, we found that tumor Apelin was associated with lymph node metastases.

A previous study suggest that tumor patients had higher Apelin levels compared with healthy controls, and Apelin is closely related to the disease stages and progression independently of other potential confounders [30]. Apelin was expressed in cultured lung cancer cell lines both at the mRNA and protein levels [30]. We observed similar phenomena in cultured GC cell lines. Increased Apelin protein level is associated with elevated microvessel densities and predicts poor overall survival, suggesting Apelin as a novel angiogenic factor in human lung cancer cell [31]. In our study we observed that GC patients with strong Apelin staining had significantly shorter overall survival period compared to those with low Apelin expression and those with weak or negative Apelin staining.

Several limitation should be addressed it this study. Firstly, the sample size is relatively small and only Chinese patients were enrolled. Secondly, the signal pathway under which Apelin/APJ pathway affects cellular biological behavior of gastric cell lines was not included in this study.

## Conclusion

In the present study, we reported that tissue Apelin status, rather than serum Apelin level, is closely associated with clinical features and prognosis of GC patietns. in vitro study indicated in GC cell lines inhibition of APJ reduced cellular proliferation rate, migration and invasion ability in vitro, suggesting the involvement of Apelin/APJ pathway in GC progression.

## Abbreviations

BMP: Bone morphogenetic protein
FBS: Fetal bovine serum
GC: Gastric cancer
MMP: Matrix metalloproteinases
OS: Overall survival
TNF-α: Tumornecrosisfactor-a
VEGF: Vascular endothelial growth factor
